# Globalization and its methodological discontents: Contextualizing globalization through the study of HIV/AIDS

**DOI:** 10.1186/1744-8603-7-29

**Published:** 2011-08-23

**Authors:** Garrett W Brown, Ronald Labonté

**Affiliations:** 1 Department of Politics, University of Sheffield, Sheffield, England, UK; 2 Institute of Population Health, University of Ottawa, Ottawa, Ontario, Canada

## Abstract

There remains considerable discontent between globalization scholars about how to conceptualize its meaning and in regards to epistemological and methodological questions concerning how we can come to understand how these processes ultimately operate, intersect and transform our lives. This article argues that to better understand what globalization is and how it affects issues such as global health, we must take a *differentiating approach*, which focuses on how the multiple processes of globalization are encountered and informed by different social groups and with how these encounters are experienced within particular contexts. The article examines the heuristic properties of qualitative field research as a means to help better understand how the intersections of globalization are manifested within particular locations. To do so, the article focuses on three recent case studies conducted on globalization and HIV/AIDS and explores how these cases can help us to understand the contextual permutations involved within the processes of globalization.

## Introduction

Academics generally add the suffix 'ization' to a word when they wish to denote that something is a *process*. When customary law becomes codified positive law, we call this a process of constitutionalization. When pressing issues are increasingly framed in terms of a security threat, we call this a process of securitization. Consequently, when we refer to increasing interconnections between peoples, economies, cultures, governments, environments and other various networks at the global level, we call this a process of globalization. In terms of etymology, globalization literally refers to the idea of 'global process' and the term originally surfaced in the late 1960s as a conceptual device to help us better understand the growing perception that the world was becoming increasingly interconnected economically, financially, technologically, culturally, politically and environmentally. As it is generally defined in any standard dictionary, the term globalization refers to 'an umbrella concept that seeks to capture the growing interconnectedness and integration of human society at the planetary scale [1] [p.112].'

However, as with any word ending in 'ization' there remains considerable discontent between globalization scholars about how to conceptualize its meaning and in regards to epistemological and methodological questions concerning how we can come to understand how these processes ultimately operate, intersect and transform our lives. In trying to provide answers to such questions, most scholars have traditionally engaged in the study of globalization from a methodological standpoint that generally relies on two foundational approaches: locating one feature of globalization as representing its defining property [2-4]; and approaching the study of globalization from the assumption that it is an epistemologically understandable phenomenon about which we can reach definite conclusions as to its processes [5,6], its positive or negative effects [7-11], its transformative capacity [12-14,6] or its irrelevance [15,16].

While yielding important knowledge claims about globalization, traditional approaches to the study of globalization often fail to capture many facets involved within its multifarious and complex processes: that whatever globalization is, it is not something that is easily definable or reasonably encapsulated by a single trend (or bundle of trends) associated with global interconnection. It is more appropriate to think of globalization as a pluralistic phenomenon with indeterminate idiosyncrasies [17,18] and anomalistic permutations [19]. As Daniel Cohen contends, there is a key dialectical dimension to globalization that is often missed by traditional approaches and these contradictions are illustrated simply by the fact that the concept of globalization has 'altered people's expectations more than it has increased their ability to act  [20] [p.166].' Methodologically, there has been a tendency for scholars of globalization to focus largely on macro-level quantitative research in order to illustrate how the world is becoming more or less interconnected. These macro-level models are largely preoccupied with the analysis of economic trends, usually ignoring other key aspects involved with the processes of globalization. They also suffer the limitation of being stylized regressions which may (or may not) inform us of the causal direction of the correlates under investigation, and little about how the effects of these trends are distributed within national populations. Although macro-level methods remain useful for locating many interconnected networks involved with globalization, they are weak in explaining how these processes are experienced or understood by those involved, bypassing idiosyncratic interfacings that take place between the global and the local.

In response to these limitations, some researchers have argued that 'when thinking about globalization we need to pay closer attention to how its numerous flows and process are encountered and informed by different actors and agencies in a range of cultural, political and social contexts [21] [p.1].' To better understand what globalization is and how it affects our lives, we must take a *differentiating *approach, which focuses on how the multiple processes of globalization are 'encountered and informed by different social groups' and with how these encounters are experienced within particular contexts [21] [p.139]. It is thus increasingly important to incorporate a qualitative and ethnographic component to globalization research to better capture its multidimensional, contradictory and at times empirically and conceptually indeterminate processes.

As a means to engage with this approach, this article examines some heuristic properties of qualitative field research as a means to help better understand how the intersections of globalization are manifested within particular locations. Its focus is on three recent case studies on globalization and HIV/AIDS and explores how these cases can help us to understand the dialectical permutations involved within the processes of globalization.

### The Conceptualization of Globalization

Before examining a contextual approach to the study of globalization, it is useful to provide further discussion about how globalization has been traditionally conceptualized and studied. Borrowing from the typology provided by David Held et al. [22], it is possible to locate approximately three traditional approaches involved with how globalization has been conceptualized: *globalist, sceptic *and *transformationalist*. Presenting globalization theory in these general terms will not fully capture the nuance, difference and sophistication of argument contained within each category. The typology is nevertheless useful in that it provides a heuristic device that can help us understand the general themes associated with various approaches in the study of globalization as well as highlight the various ways scholars see the processes of globalization impacting upon our lives.

To begin, a *globalist *approach (also sometimes referred to as *hyperglobalist*) to the study of globalization tends to conceptualize globalization as a progressive process of market interconnectedness that is ushering in a radically 'globalized' era in human history. This new global epoch is constituted by a systematic breakdown of economic barriers, of sovereign borders, denationalization and of traditional ideological disagreements involving economics and politics. From this 'globalized' ontology, most globalists view the process of globalization as a positive expansion of the global market, which is supported by an exponential growth in economic transactions [5], trade [23], foreign investment [24], labor mobility and the growth of multinational corporations [25]. Because of this increased economic integration, some scholars like Kinichi Ohmae maintain that global capitalism holds the promise of creating global interdependence to the point where territorial boundaries and state sovereignty are weakened in favor of a more economically, politically and culturally unified world [7]. Other scholars have preferred to focus on the inherent qualities of global capitalism in order to suggest that globalization represents a process of progressive global economic advancement and that market capitalism has both the capacity to create global prosperity as well as the ability to overcome existing negative externalities [6,26]. What is common within the globalist position is the conceptualization of globalization as an economically driven process that is moving humanity closer to a more unified world, one in which the disparities between borders, markets, economies and cultures are radically reduced in favor of a more common global condition.

Conversely, if the globalist argument is one that roughly perceives globalization as a process of progressive economic and liberal ideological advancement, then it is possible to understand a *sceptical *approach as one that challenges the positive elements involved with this globalist reading. However, to be clear, the sceptic position is a fairly broad church and it maintains roughly three sub-categories. First, many sceptics believe that globalization is an actual phenomenon and that it encompasses several economic, political and cultural dimensions that have profound impacts on people's lives. Nevertheless, sceptics disagree with globalists about the positive 'nature' of globalization (at least its economic dimension and the politics that support it), arguing that the benefits of globalization are largely restricted to, or asymmetrically skewed towards, the three trading blocks of North America, Europe and Japan [8]. Because of this, sceptics view globalization as something of a misnomer and believe that it is more appropriate to speak of globalization in terms of triadization [8], globalism [27], exploitation [28,29], dependency [30], neo-liberalism [10,31], neo-imperialism [32,33] or simply the global diffusion of capitalism with its traditional crises of over-production/under-consumption compounded by the increased financialization of global markets in which currency and investment speculation affords more opportunity for capital accumulation than participation in the 'real' economy [34]. Second, some sceptics have in the past challenged the use of globalization as a conceptual device by arguing that globalization is nothing more than 'a term used by a lot of wooly thinkers who lump together all sorts of superficially converging trends [16]' and that it is so conceptually impoverished as to 'not even have existed [15].' In this regard, some sceptics have suggested that globalization is an over-hyped and over-inflated concept in the social science lexicon that has little analytical and social scientific purchase, at least as a wholly new or distinct phenomenon. Others have argued that different constructions of globalization are leading politicians and businesses to act *as if *the globalized world exists as socially imagined, and in doing so are actively creating that to which they assume they are only responding [35]. Waldon Bello, a Marxist-inspired sceptic of globalization's economic inequalities, is one of a number of writers who argue for a 'deglobalization.' Unlike more extreme localists [36], Bello acknowledges the importance of international economic engagement and exchange, but calls for a deliberative reorienting of national economies from an emphasis on export for foreign markets to production for domestic consumption; a strategy that China is, in fact, now pursuing to overcome the decline in its US and European export markets. Illustrative of the blurring between these three typologies, however, Bello argues the necessity of a pluralist system of global economic governance in order for national economies to successfully de-globalize [37]. Lastly, some sceptics, as if to reinforce Bello's point, have presented a 'post-globalist' challenge to globalization, by suggesting that globalization represents a dying phase in human history that is 'unexpectedly over [14]' and that globalization has been replaced with a new era of reinvigorated nationalism and religious fundamentalism [10].

The third conceptualization of globalization focuses less on locating a specific 'driver' involved with its processes and, instead, it attempts to locate the various ways in which globalization symbolizes an unprecedented period of global interconnectedness and social *transformation*. In this regard, globalization is not inherently good or bad, or primarily related to a single global impetus, but is seen as the result of incalculable economic, cultural and political 'transformations' that are 'restructuring the ways in which we live... in a very profound manner [38].' According to Anthony Giddens, these transformative processes are intrinsically associated with conditions of modernity in that contemporary social systems are increasingly 'stretched' across territories, identities, economies and cultural life-worlds [39], even if huge asymmetries in the pace of, control over and benefits of such 'stretching' persist. Because of this spatial-temporal *distanciation*, globalization has come to affect every dimension of contemporary life from the creation of new global narratives regarding economic integration to more mundane aspects of everyday living. Although many of the world's peoples, particularly in low-income countries, are marginal actors in such processes, they can be dramatically affected by them, as in the disproportionate burdens of poverty and unemployment experienced by many poor countries in the wake of the 'first world' global financial crisis of 2008. Consequently, many transformationalists believe there are compelling arguments offered by globalists as well as sceptics and that any study of globalization will deliver evidence to support both positions [22]. Therefore, as an alternative, the transformationalists try to navigate a middle position by focusing on how the processes of globalization are changing our social structures and to examine what negative and positive impacts these processes have when they further 'stretch' social relations beyond traditional borders. The global diffusion of digital technologies, as one example, permits the global market integration economies favored by globalists; while creating new forms of oppositional social mobilization by those sceptics who focus on the negative externalities of such integration. Unlike the sceptics and globalists, transformationalists do not conceive globalization as inherently good or bad, or as being primarily economic or cultural.

As this brief sketch illustrates, there is little consensus between globalization scholars about what globalization is and about what impacts it ultimately has on our lives. At a minimum, apart from a few sceptics, there is agreement that the definition of globalization involves the recognition of some form of greater integration and interconnection at the global level. Furthermore, there is also fairly widespread consensus that the new language of globalization is necessary to help describe geographical and political issues of denationalization, the diminution of the significance of territorial boundaries and deterritorialization [40]. However, as Manfred Steger points out, this broad definitional agreement and use of common language remains extremely abstract and 'tells us little about [globalization's] remaining qualities [2].' These conceptual difficulties are compounded by the fact that there is widespread disagreement about what key processes or properties underwrite globalization. In this regard, not only do globalization scholars disagree about how to understand the 'nature' of globalization on a meta-theoretical level, but there are also fundamental disagreements about what empirical processes (economic, cultural, political, territorial) should be studied as a means to try and define its character and its effects. Furthermore, as will be discussed below, there is also discontent about what research methods are most appropriate to 'capture' the empirical characteristics of globalization and how to best incorporate this methodology as a means to substantiate any one particular conceptualization of globalization. The key point is that the following methodological orientations often arise directly out of how globalization has been conceptualized, which then transfers existing conceptual disagreements onto debates concerning what empirical properties constitute the processes of globalization and what methodological tools are best suited to capture these processes.

### Methodological Traditions to the Study of Globalization

Four methodological traditions are discernable in the globalization literature. First, as alluded to in the Introduction, most scholars have tried to locate one dominant trend involved with the process of global interconnectedness and to then single this aspect out as representing its primary defining property. The focus has largely been on global economic and financial trends, isolating this feature of growing interdependence as the key empirical driver behind how we should study as well as conceptualize globalization. Although there are scholars who continue to defend the significance of cultural globalization [19,41], technological globalization [42,43] and political globalization [40,44,45], for the most part, and largely en-masse, mainstream globalization scholars have tended to reduce these other processes as 'derivable' or as 'satellite externalities,' arguing that 'economic globalization has been the driving force behind the overall process of globalization over the last two decades [46].' This has been applied to the study of globalization and health, as much as it has to other sectoral concerns [47,48]. Although the global economy is clearly a powerful driver involved with whatever globalization is, this does not mean that everything can be reduced to economic argument. Equating globalization solely with processes of economic integration or with a particular (neo-liberal) vision of market capitalism inevitably sidesteps key sociological and historical aspects involved with globalization that are transforming our social life-worlds in culturally and politically sensitive ways. What is often missing, and what will be explored in the next section, is a multidimensional approach to the study of globalization that, while incorporating its economic dimensions, can also help to make sense of the many localized, idiosyncratic and pluralistic qualities.

Second, most globalization scholars focus largely on macro-level analysis relying on quantitative data and longitudinal economic analysis to prove how converging trends are creating interconnection and integration (or not) at the global level. Of course, globalization by its very etymology suggests a macro 'global process,' implying that the essence of our understanding should move from the global to the local. In this way, the study of globalization necessarily requires an element of macro-level quantitative analysis in order for us to understand many of its large-scale processes and as a means to help explain how these transform issues of global cohabitation. Nevertheless, to say that macro-level quantitative analysis is a *necessary *component to understanding globalization is not to say that such analysis is by itself *sufficient*. This is because macro analysis is best suited for locating global trends, broad global interconnections and large-scale networks, but is insufficient in telling us about how these phenomena specifically affect local communities or how these trends are interpreted and encountered by various social groups and communities. We recognize that the same limitation applies to national or even sub-national population studies that rely upon large-scale survey or panel data; indeed, our arguments for a broader approach to globalization studies that use both quantitative and qualitative/ethnographic data and methods have long been made for social science studies at lower levels of aggregation [49,50]. Therefore, as the next section will examine, it is also useful to supplement macro-level research with more micro-level contextual qualitative analysis that can help to capture processes of 'glocalization', the intersubjective transformations that take place at the interface between the global and the local [51].

Third, there has been a strong tendency by globalization scholars to adopt a deductive methodological approach. When surveying the literature on globalization, it is common to see studies that start by producing a theory about what specific and isolated 'global process' is key to understanding globalization and to then generate a hypothesis about how this variable can confirm a particular positive or negative effect. As with all social scientific research, the generation of this hypothesis is usually based on a pre-formulated theoretical conceptualization. From this theoretical frame we then judge the worthiness of this conceptualization by how well it can explain a particular phenomenon and by how well it can 'make sense' of seemingly disparate empirical experiences. To be clear, deductive reasoning is an extremely useful research method. There is also no reason to suggest that deductive reasoning must remain mutually exclusive of a more inductive approach, in which analyses of observational data (fieldnotes, interviews, focus groups, texts and other qualitative sources) lead to novel insights about social phenomena that may be transferable (generalizable) and that can build new or challenge/nuance existing theories. What remains troubling about a good deal of research on globalization is that until recently it often failed to successfully incorporate a more inductive element within its overall research design, thereby lacking in applied and theoretical relevance when transposed to localized contexts.

Lastly, many studies of globalization fail to properly capture and express the dialectical features associated with globalization. This is because globalization is seemingly both good and bad; it is dialectical in the Habermasian sense in that it often presents both a thesis and an anti-thesis [52]. One thesis, for example, holds that globalization promotes more interconnectedness, resulting in greater economic markets, the spread of democratic values, and cooperation on issues of global interdependence; while these same processes simultaneously produce an anti-thesis of greater economic inequality, ideological tension and an overall failure to secure human development. Although this is less of a problem for transformationalist approaches, globalists and sceptics have tended to focus narrowly on a single trend associated with globalization in order to come to a definitive judgment as to whether globalization represents a good thing, a bad thing or a mythical aberration. Therefore one problem with this approach is that globalization can often reflect all of these properties simultaneously and dialectically, a point we return to in the conclusion to our article.

The argument presented here is not meant to suggest that more traditional approaches to the study of globalization have necessarily failed to respond to the concerns listed above. Burawoy and colleagues, as one example, are sceptics who argued for, and undertook field research based in the experiences of globalization's constraining economic effects on the lives of diverse peoples in different parts of the world [53]. Neither should this article be viewed as advancing the sceptical position which suggests that the study of globalization represents nothing more than a bunch of 'superficially converging trends' with little social scientific value. On the contrary, the suggestion is that we need to supplement these traditional approaches with a more inductive research agenda that is open to the complexities involved with globalization, especially as they are encountered in particular contexts; that is, one that embraces many of the conceptual aspects of the transformationalist position. This requires adoption of a multidimensional, inductively driven and contextualized approach that builds upon qualitative fieldwork and local ethnographic observation in order to help connect localized experience 'upwards' to various global trends (economic, political and cultural). It is in the next section that an approach of this kind will be explored in relation to three recent case studies conducted on globalization and HIV/AIDS.

### Contextualizing Globalization Through Studies in HIV/AIDS

The three case studies involved with the remainder of this article are part of a larger project on Economic Globalization and HIV/AIDS conducted in association with the IDRC and HEARD at the University of KwaZulu-Natal, South Africa. Our goal here is to highlight some of the ways that these qualitative and ethnographically driven field studies improve our understanding of what globalization is and with how processes of globalization can transform particular social contexts and activities pertaining to HIV/AIDS.

#### A) Global Transformations of Transactional Sex in Mbekweni Township South Africa

The first study conducted for the HEARD/IDRC project involved the examination of economic globalization and transactional sex practices in Mbekweni Township in South Africa. Transactional sex refers to the exchange of gifts (material, monetary) for sex framed outside of prostitution or sex work by those who participate in the exchange.

The study focused on transactional sexual behavior of young women between the ages of 16-24. The study included on-site ethnographical observation as well as interviews conducted with community leaders, focus groups, the young women involved with the sexual behavior as well as with the men who provided goods and services as transaction partners [54]. The rationale behind the study was to help locate some of the key underlying factors that drive peoples' pursuit of transactional sex and to inductively explore in what ways these factors could be connected to, or derived from, processes of globalization.

The overall findings of the study demonstrated that involvement in transactional sexual risk behavior was multidimensional and transformative in that it connected to the processes of globalization in both economic and cultural ways. In particular, the research indicates a strong connection between female aspirations for a modern lifestyle in line with popular images of global modernity, and a rationalization to satisfy the material basis for obtaining this image even at an increased risk for HIV infection through transactional sex. Through a blend of economic and cultural influence, various images provided 'global windows' or 'Westernized dreams [55]' to a promising ideal of modernity, of material success and an enhanced 'globalized' social status. The problem, however, as Daniel Cohen argues, is that 'for the majority of the poor inhabitants of our planet, globalization is only a fleeting image... what we too often ignore is how strong this image is, how pregnant with promises yet to be fulfilled [20] [p.6].' In this regard, and in the case of the Mbekweni women studied, 'globalization creates a strange world where... it has altered people's expectations more than it has increased their ability to act [20] [p.166].' In the case of those participating in transactional sex within the township of Mbekweni, it is possible to locate three multidimensional transformative features that have altered their expectations.

First, in the past, the study of transactional sexual behavior suggested that young women engaged in this activity as a means to secure basic needs and to obtain the material goods necessary for more immediate survival. This sexual behavior has been linked to the increase of unsafe sexual practices, a lack of condom use, and increased substance abuse, multiple sexual partners, physical violence and the spread of HIV [56-58]. However, as the Mbekweni study shows, there is increasing evidence to suggest that motivations for young women's involvement in transactional sex is not confined solely to matters of sustenance but include women's pursuit of 'material goods for pleasure and vanity [54]' as a perceived 'gateway' to modernity [59,60]. As the Mbekweni study further finds, 'the pursuit of money and material goods was emphasized as being of great importance, far outweighing fears of HIV infection [54].' Thus, the meeting of basic survival needs was not the only primary motivation for high-risk sexual behavior; or, alternatively, one could argue that globalized images of modernity through material acquisition had subtly but pervasively altered these women's perceptions of what needs should be considered basic and what constituted meaningful survival. In addition, many of the women involved within the study considered themselves as 'hunters' who were engaged in transactional sexual acts as a means for social and material mobility. In order to obtain certain items of value, the young women would actively seek out men who had money or who were perceived to have a high level of social status and material sophistication [54].

Second, the social indicators of whether a male satisfied this image of material and social status were measured largely by globalized Western symbols of modern wealth and prosperity. For example, many women involved within the study specifically discussed how they were going to 'get the one driving the yellow Kompressor' Mercedes Benz and how a car like that symbolizes the right kind of 'sugar daddy.' This preference for seeking global icons of material wealth and success was also evident with male participants and often concerned more mundane items such as the type of beer one drinks. As one participant suggested, 'if I am the boss I'll be drinking Heineken. I will buy a Heineken, you see... then a second one, so that she'll notice that [name of participant] has a lot of money on him.' Again, like the Mercedes, the symbol of wealth and sophistication was often associated with an imported item that had a certain level of global status, which in the case of this one male participant, was seen to be the most successful means to obtain a sexual partner [54].

Third, the material goods sought when engaging in transactional sex by the female participants also reflect an aspiration to pursue items that capture the global image of modernity. In many cases, women were engaging in transactional sex in order to buy clothing from relatively cheap local South African retail stores such as Mr. Price and Chinatown outlets. These particular retail stores specialize in providing cheap knock-off clothes that capture the look and styling of the more famous designer products of Europe and the United States. The women who participated in the study emphasized the need to have a large selection of clothing and indicated a preference for quantity over quality. What is most interesting about this is that it supports Cohen's argument that the processes of globalization create a dialectical relationship between global imagery, product desire, market availability and economic dependency. Or more appropriately, it illustrates that despite being able to pursue items that resemble the global imagery of high-end fashion by engaging in transactional sex, the actual products that are available or within the economic reach of those involved are only cheap knock-offs. At the same time, for many of these women (and for those in the Madagascar study as well, described later) the global 'labeling' of clothing was less important than having a large quantity of 'the right look.' What had become globalized was a capitalist modernity of excess consumption, in which women didn't simply want high fashion, regardless of label, but closets-full of clothes they could change into a half dozen times a day. In addition, and in order to tie this relationship more broadly into features of market globalization, these clothes are mostly produced by large multinational Chinese textile and garment firms in the Far East and Lesotho and are only available at a low cost due to the poor quality of the product. As Cohen would suggest of this relationship, global images are dominant, but local economic restrictions persist.

The transformations associated with these multidimensional and dialectical qualities have significant implications for how we understand the relationship between the processes of globalization and HIV/AIDS. The Mbekweni study found that money and the procurement of material goods was of a 'greater priority than ensuring safety against HIV [54].' It further suggests that part of this behavior is due to an 'over-emphasis on clothing and consumerism in general, through global communications that glamorize fashionable images of beauty and success [54].' This over-emphasis, and its creation of new forms of 'social necessity,' may have led in this case to poor quality purchases. But it remains a product of globalization's economic underpinnings: that is, the aggressive pursuit by transnational corporations through investment and advertisement of new consumers in new markets, aided by liberalized trade and investment treaties, to offset declining rates of profit in already saturated higher-income countries [47]. In addition, the study found a growing fatalism among the female participants, where the stigma of HIV/AIDS had faded, where the virus was likened to a brand of perfume that is popular in the community. This suggests a certain level of normalization of HIV/AIDS in relation to high-risk sexual behavior and the legitimization of transactional sex as an appropriate means to satisfy material desires. As one female participant suggested, 'it doesn't matter if he's sick... as long as he satisfies your needs for money and alcohol, you don't really care [54].' Transactional sexual behavior is not a product of globalization and has certainly existed as long as there has been written history; but as the case of Mbekweni illustrates, the processes of cultural and economic globalization have transformed the social dimensions of how transactional sex is practiced, with serious implications for interventions intending to prevent HIV/AIDS.

#### B) Global Modernity Meets Traditional Resistance: Malagasy Youth

The second case study set out to understand the relationship between an interest in the consumption of designer goods and young people's sexual behavior in two regions of Madagascar: Antsiranana, a city in the north, and the capital city Antananarivo along with its peri-urban and rural outskirts. This study was motivated by former ethnographic research in the country that pointed to urban youth using transactional sex as a means by which to access images of modernity [61]. Specifically, this past work examined youth in the coastal city of Tamatave and made links between an increasing importance placed on consumption (and transactional sex as a means to consume) to the liberalization of Madagascar's economy. Furthermore, this research suggested that transactional sexual practices, particularly among young urban Malagasy women and foreign men, were playing a significant role in social class formation, 'engender[ing] new hierarchies associated with globalization and neoliberal economic reform [61] [p.574].'

Inspired by this former research, the Madagascar case study used both qualitative and quantitative approaches to explore how youth (15-24) understood the symbols of modernity as well as to capture the extent to which they perceived a relationship developing between sexual behavior and the increased premium placed on accessing these symbols of modernity. To examine this relationship, the project relied upon the use of focus groups, a structured household survey and in-depth interviews.

The findings from Anatananarivo support a more transformative understanding of how globalization may be influencing behavior and capturing the imaginations of Malagasy youth. One of the concepts explored in detail was that of '*lamaody*,' which in the context of Antananarivo, the capital city and its environs, is used both as a noun and an adverb to describe ones relationship with 'modern' living. From the French 'la mode' (France having been the colonizing country), *lamoady *refers both to the material items of modernity in the form of fashionable clothes, technologies and accessories; and to a lifestyle which is marked by consumption of alcohol and cigarettes, having multiple sexual partners, using the internet and other new technologies, and consuming media in the form of music and films from abroad. *Lamaody *was often referred to as originating from outside of Madagascar; and those who were able to keep up with it were described with both admiration and contempt.

The very word, *lamoady*, captures a dialectical aspect of globalization, for it signifies a meaning of global progressiveness as well as a condition of poor judgment over how one achieves it. On one hand, the strong Christian moral values on women's chastity in Antananarivo (arising from an earlier colonial epoch of globalization and pre-colonial religious conversion) temper the draw toward the imagined modern life. The notions of these foreign values are considered dangerous, and as one participant explained, went 'against the norms [62].' Consequently, unlike the case in Mbekweni, transactional sex in Antananarivo is not (yet) a commonly reported practice, nor is it deemed an acceptable strategy toward achieving a modern lifestyle. On the other hand, and at the same time, it was often described as one of the few reliable means by which young people, particularly women, can obtain the symbols of modernity. Because most people have to resort to such 'immoral' practices in order to touch the 'fleeting image' of modernity, the image becomes a tainted one 'pregnant with promises yet to be fulfilled [20] [p.166 & 6].'

This duality led to a complicated understanding of whether young women who followed *lamaody *were to be respected or shamed and represents an interesting permutation of 'glocalization.' On the one hand, *lamoady *was characterized in harshly negative terms: that Western influence with its emphasis on materialism and a consumer culture was associated with decaying morals and what were deemed as venal interests, captured by terminology depicting individuals as '*sipa maté*' (young materialistic woman) or '*bandy maté*' (young, materialistic man). On the other hand, for those young women engaging in transactional sex for *lamoady*, there was also shame in not capturing aspects of this material world: appearing poor and shabbily dressed, reflected badly on the community from which she came. As one participant suggested, 'in our building, you'd feel ashamed if you wear a torn sandal and mended slacks [62].'

At issue may be the scale of consumption and the surrender to the globalized mythos of 'modern living' through the acquisition of goods beyond those considered socially necessary. The notion of being a '*materialiste*' was unambiguously negative for young men and women in Antananarivo. A *materialiste *was someone whose primary motivation in pursuing sexual relations is to access money or goods. Once again, a foreign term is used to describe a behavior that is seen largely as foreign-inspired and driven by the desire to obtain foreign goods. Here the negative emphasis was on one's motivation for seeking partners. In interviews with male university students, though more broadly representative of youth opinions, women who 'lay down their body...for the love of *lamaody' *were contrasted with women who engage in exchange relationships for their livelihood (e.g. to obtain school fees) [62]. As a result, most interviewees held a high level of tolerance for those engaged in transactional sex when it was used toward their children or self-improvement (i.e. education). As one young man put it, 'if the girl does that only for *lamaody*, it's not at all acceptable...if she were doing it to make a living, then I wouldn't mind [62]'.

#### C) Global Transformations of HIV/AIDS Funding and Public Policy in Peru

A third study conducted for the HEARD/IDRC project involved an examination of the relationship between global HIV/AIDS funding mechanisms and the development of public health policy in Peru. The study involved the collection and analysis of health budget information, a content analysis of official health policy documents and key informant interviews with social actors, health authorities, non-governmental organizations and affected communities [63]. As with the previous studies, a key research question of the project was to explore the various ways the formulation and practice of HIV/AIDS policy in Peru was connecting to, and being influenced by, processes of globalization.

Similar to the Mbekweni case, the results of the Peruvian study demonstrate that the relationship between HIV/AIDS public policy formation in Peru and the processes of globalization are multidimensional and involve more than just economic processes. It also highlights a contextually specific property of 'glocalization' in that the influence of global health funding mechanisms was effectively amalgamated into Peruvian policy in uniquely national terms. The complexities of this particular mixed-methods case study are discussed in more detail elsewhere [63]; we focus here on how this study helps to demonstrate three of the multidimensional and transformative characteristics of globalization associated with global politics, economic trends and the cultural expansion of globalized health rights terminology.

First, the Peruvian study highlights a key process of political globalization and the ways global governance mechanisms intersect with localized HIV/AIDS policy formation and implementation. Although Peru had established prevention-related programs prior to 2000, it was not until Peruvian involvement with various global health initiatives such as UNGASS and The Global Fund to Fight AIDS, Tuberculosis and Malaria (GFATM) that greater resources and political mobilization were directed toward HIV/AIDS treatment. This suggests the existence of a correlative relationship between local policy formation, the influence of global governance processes, and available access to global 'seeding' money for ramping up localized initiatives. Peruvian engagement with GFATM funding policy also transformed a primary HIV/AIDS governance mechanism and how HIV/AIDS related policy was formulated and funded. As the study outlines, part of the GFATM funding grant stipulated that the Peruvian government would make certain political and economic commitments to the formation and implementation of HIV/AIDS policy. For one, the grant application process of the GFATM required the establishment of a multisectoral Country Coordination Mechanism (CCM) to facilitate local stakeholder participation. The study showed a direct link between GFATM CCM policies and the formation of a Peruvian CCM mechanism that was eventually incorporated into its overall health governance system. The GFATM grant was designed to cover ARV costs for the first two years with a commitment by the Peruvian government to ramp up their coverage of those costs thereafter. The Peruvian government, the CCM grant design mechanism and the Technical Review Panel of the GFATM considered the grant as a form of seed money for establishing a sustainable and purely internal Peruvian health treatment policy for HIV/AIDS.

Second, there was an infiltration of the expanding global language about stakeholder participation and a transfer of 'health rights' terminology into Peru's general discourse on health policy. As the study indicates, the global HIV discourse on health rights has started to 'expand to other diseases, primarily tuberculosis.' In this regard, much of the global language around HIV mobilization initiated by social movement activists in regions as disparate as South Africa and the USA has helped to create a standardized benchmark in regards to the defense of health rights of affected people and against stigma and discrimination. In limited ways this language has increasingly been incorporated into other policy discussions involving Peruvian health professionals, civil society movements and some officials. However, in terms of actual multisectoral participation, only the GFATM programs on HIV and tuberculosis have produced procedures to 'officially recognize the participation of community based organizations [63].' One possible explanation for this isolated infiltration of participatory governance is that GFATM policy provided a strong global 'steer' for accelerated inclusion of multisectoral governance in the case of HIV/AIDS and tuberculosis; and that 'it was difficult for people affected by other diseases to reach the achievements of HIV mobilization.' Many interviewees considered 'such response is unlikely for other diseases that will not have this kind of support: a global movement, international interest and large resources [63].'

This finding suggests an interesting dialectical relationship between GFATM policies and how they affect local mobilization for other diseases. Engagement with the GFATM has established a double-edged condition: the increase in attention and funding for HIV/AIDS has had tangible positive effects in terms of providing free ARTV treatment and in insuring multisectoral participation, but has simultaneously 'crowded out' other diseases in terms of generating a similar mobilized response. One implication of this dialectic feature is that 'HIV mobilization achievements challenge the concepts of equity and access in health, since the Peruvian government does not ensure access to free treatment for other diseases [63].' Interviews conducted with non-HIV/AIDS related NGOs saw this condition as 'unfair'. In other words, the discrimination now favoring HIV/AIDS in Peruvian health policy could form the basis of challenges for greater equity in health care access across a wider range of disease conditions.

Third, although the seeding money from the GFATM provided an initial financial and political motivation to formulate distinct health policies for the treatment of HIV/AIDS, it was largely due to Peru's economic growth that treatment expansion was sustainable. As the data suggest, in a context of economic expansion, with increased tax revenues enabling great fiscal expending from public resources, the Government of Peru was able to replace the withdrawal of resources from the GFATM, without affecting expending on other health programs. It was able to do so without a diversion from other health expenditure categories, suggesting that, in this particular instance, the sceptic concern about reliance on development health aid and sustainability 'drop-offs' after external funding evaporation was not a prevalent feature. This was primarily due to Peru's strong economic growth during the same period (something not necessarily guaranteed into the future) as well as to the political motivation to maintain treatment targets. Although it is not possible to generalize from this particular case, the Peru study does highlight an interesting feature involved with GFATM funding policy in national economic situations where capacity development may have a stronger chance of success. Unlike in many relatively poor economic countries, where debt management and high levels of HIV/AIDS infection cripple mobilization and capacity building efforts, Peru's economic growth and relatively small pandemic numbers allowed it to effectively sustain a near universal treatment and comprehensive care program.

This is not to say the globalization in other of its processes is devoid of health effects, whether positive or negative. A 'sceptic's' one-year review of the Peru-US Free Trade Agreement (FTA), which came into effect February 1, 2009, documented a number of rapidly declared decrees that failed to strengthen (as promised) labour rights but succeeded in weakening environmental and land ownership protections to facilitate more rapid exploitation of natural resources of interest to American investors [64]. Both outcomes are more likely to worsen than improve health inequities in Peru. The FTA has led initially to lower drug prices on US imported products, attributed to elimination of an international tax previously levied by Peru and increased retail competition as international drug store chains expand across the country [65]. Drug prices nonetheless could eventually rise as extended intellectual property rights in the FTA begin to affect patented drugs, increasing the costs of providing ARVs and, importantly, treatments for diseases with advocacy groups which currently look with some resentment to the special arrangements so far won in treatment for HIV/AIDS [66]. What the Peruvian study nonetheless reveals is that processes of political, cultural and economic globalization can be conceptualized as having negative as well as positive effects. Furthermore, the discovery of these idiosyncratic qualities are best teased out and examined through an inductive and contextually focused approach that utilizes qualitative and ethnographic methods. Because of this, like the Mbekweni and Antananarivo studies, the Peru study is able to locate striking idiosyncratic and dialectical features involved with globalization while generating interesting new questions and complex implications about how we might better understand the relationship between global process and HIV/AIDS. In this regard, by incorporating a more differentiated and contextual approach, these studies are able to supplement as well as compliment more macro-level quantifiable studies and to generate further insights about how globalization operates at both the global and local level.

## Conclusion

### The Importance of Understanding Globalization in Context

This article began by suggesting that the study of globalization required a more contextual approach that could better capture the transformationalist dimensions of how the processes of globalization operate. To support this argument, this article examined three traditional approaches to the study of globalization and highlighted some of the conceptual and methodological shortcomings that are generally involved with these efforts. In order to understand globalization and how it specifically impacts upon our lives, it is increasingly important to study globalization in terms of how its processes are encountered by various social groups in different social contexts. To do so, it was suggested that the study of globalization needed supplementation with more contextual, inductive and qualitative approaches.

To demonstrate how this approach could operate in practice, this article examined three recent case studies that attempted to make 'bottom-up' links between local HIV/AIDS issues and globalization. These contexts provided interesting and implicative insights into the multidimensional ways that globalization intersects with local social structures and how these processes effectively 'stretch' social structures. In all three instances, globalization was a dialectical phenomenon that, depending on the context, illustrated positive as well as negative qualities. In the case of Peru, this manifested itself through the empirical fact that near universal ARV treatment was now available in Peru and that through a combination of local effort and external globalized mechanisms Peruvian programs were sustainable and effective. However, at the same time, there was evidence to suggest that these mobilization efforts also had negative effects in regard to equitable funding mobilization for non-HIV/AIDS related diseases and that there were indications that various participatory governance mechanisms involved with HIV were not transposed onto other health risk policy debates.

Likewise, in the case of Mbekweni, a blend of multidimensional elements fused together to create some unique HIV/AIDS policy implications: limited economic avenues in conjunction with powerful globalized consumer images created idiosyncratic transformations in social sexual behavior. In this case, the heightened risks involved with transactional sex seemingly involve a *glocalized *[51] or *hybridized *[67] blend between local sociology, economic conditions, globalized expectation, new materialistic desires, and the limited ability to act upon those expectations and desires. In the context of Mbekweni, this unique blend of economic and cultural transformative process challenges prior studies that have viewed transactional sex as primarily a means for basic subsistence and survival. That basic needs may not in fact be the primary motivator (or that they have been reconfigured by globalized images to include certain forms of consumerism) has implications for both prevention and intervention strategies.

The Madagascar case presents an even more nuanced understanding of the same dimensions found in the Mbekweni case. Straddling two different epochs of globalization (the Christian morality introduced by colonialism and the desirable baubles of today's global modernity), transactional sex was acknowledged as a means to the end of *lamoady *while lamented as a symbol of moral vicissitude. But all was understood or forgiven if the woman so engaged had a 'nobler' intent than merely acquiring universalized fashion or engaging in the status power of excess consumption, such as a desire to provide for her child's education.

Lastly, this article does not provide an exhaustive study into what a differentiated and contextual approach to globalization would entail and it could not systematically explore the implications of this approach if applied more widely to global health research. In this regard, the aim of this article was not to thoroughly set out a concrete research agenda, but to draw attention to the importance of inductive methods to the study of globalization and to suggest that these approaches ought to be more seriously considered. Furthermore, this article is not meant to suggest that this is a novel approach per se, for other scholars have developed and pursued such research elsewhere. What is being suggested is that a more differentiated and contextual approach to the study of globalization should supplement traditional methods in order to help capture unique and idiosyncratic elements involved with how social transformations take place between the local and the global. And it is by looking at these intersections, that we can have a better chance of understanding what globalization is, how its processes work, and how these processes transform our lives in profound and meaningful ways - both collectively and in particular settings.

## Competing interests

The authors declare that they have no competing interests.

## Authors' contributions

GB and RL conceived the general outline of the article. GB wrote the first draft. RL revised and wrote sections of the second draft. Both authors discussed and responded to the reviewers' comments. Both authors read and approved the final manuscript.

## Authors' information

Garrett W. Brown is Senior Lecturer in Political Theory and Global Ethics in the Department of Politics at the University of Sheffield. His publications include work on cosmopolitanism, globalization theory, international legal theory and global health governance. He has recently published *Grounding Cosmopolitanism: From Kant to the Idea of a Cosmopolitan Constitution *(Edinburgh University Press, 2009) and co-edited *The Cosmopolitanism Reader *(Polity, 2010) with David Held.

Ronald Labonté is Canada Research Chair (Globalization and Health Equity) with the Institute of Population Health and Professor in the Faculty of Medicine, University of Ottawa. His research interests include the health equity impacts of contemporary globalization, health worker migration, medical tourism, health and human rights, global health diplomacy and comprehensive primary health care reform. Recent co-edited books include *Global Health Volumes 1 -4 *(Sage Major Works, 2011) and *Globalization and Health: Pathways, Evidence and Policy *(Routledge, 2009).
